# Triple malignancy in a single patient including a cervical carcinoma, a basal cell carcinoma of the skin and a neuroendocrine carcinoma from an unknown primary site: A case report and review of the literature

**DOI:** 10.1186/1752-1947-5-462

**Published:** 2011-09-19

**Authors:** Mohamed Mesmoudi, Saber Boutayeb, Tarik Mahfoud, Rachid Aasab, Nabil Ismaili, Meryem Glaoui, Hassan Errihani

**Affiliations:** 1 Department of Medical Oncology, National Institute of Oncology, Rabat, Morocco

## Abstract

**Introduction:**

The occurrence of multiple primary cancers is rare. Only a few cases and patient reviews of an association of triple malignancy have been reported.

**Case presentation:**

We report here a case of a 78-year-old Moroccan woman presenting initially with a synchronous double malignancy, the first in her cervix and the second in her skin. Our patient was treated with radiation therapy for both tumors and remained in good control for 17 years, when she developed a metastatic disease from a neuroendocrine carcinoma of an unknown primary site.

**Conclusions:**

Although the association of multiple primary cancers can be considered a rare occurrence, improving survival in cancer patients has made this situation more frequent.

## Introduction

The increasing effectiveness of cancer therapies and the improvement of diagnostic tools have led to better survival rates among cancer patients. This situation has made the problem of developing subsequent primary tumors more frequent. In the literature the prevalence of multiple primary cancer (MPC) is estimated between 0.73% and 11.7%, and the incidence is increasing with age [1].

According to the Surveillance, Epidemiology and End Results cancer registries of the National Cancer Institute, cancer survivors had a 14% higher risk of developing a new malignancy than would have been expected in the general population. Females had a slightly higher relative risk than males for all subsequent cancers combined, and the most implicated sites were breast, colon, lung and melanoma of the skin [2].

MPC is classified into two categories depending on the time of diagnosis of each primary site. Synchronous cancers occur at the same time or within an interval of two months, while metachronous cancers follow in sequence and more than two months apart [3].

Despite its low incidence, the association of two malignancies in a single patient has been widely reported in the literature, while only a few cases of three malignancies have been described. The aim of this article is to present an exceptional case of an elderly woman treated initially for a synchronous squamous cell carcinoma of the cervix and a basal cell carcinoma of the skin, who developed a third malignancy described as a neuroendocrine carcinoma from an unknown primary site.

## Case presentation

A 60-year-old Moroccan woman came to our institute 18 years ago with a history of vaginal bleeding, malodorous discharge and vaginal discomfort. Furthermore, our patient presented with a slowly enlarging skin lesion localized on the dorsum of her nose. Gynecologic examination found a 5 cm exophytic friable lesion arising from her cervix and involving the upper half of her vagina. Biopsy confirmed the diagnosis of a well differentiated squamous cell carcinoma and the absence of parametrial infiltration; neither adenopathies nor distant metastases were found. In addition, a skin inspection found a 2 cm solitary nodule on the dorsum of the nose: the lesion bled spontaneously at the examination and biopsy confirmed the diagnosis of basal cell carcinoma of the skin.

Our patient refused the surgical excision of the nasal lesion, and so treatment consisted of radiation therapy; 30 Gy external beam radiation was delivered in 10 fractions with 3 Gy per fraction over two weeks, followed by 30 Gy delivered by brachytherapy.

The cervical carcinoma was treated with radiation therapy. A whole pelvic external beam radiation dose of 40 Gy was delivered in 20 fractions with 2 Gy per fraction over four weeks, followed by 30 Gy delivered by brachytherapy. In summary, she received 70 Gy total dose radiation on the cervical cancer, and 60 Gy total dose radiation on the skin cancer.

**Figure 1:**
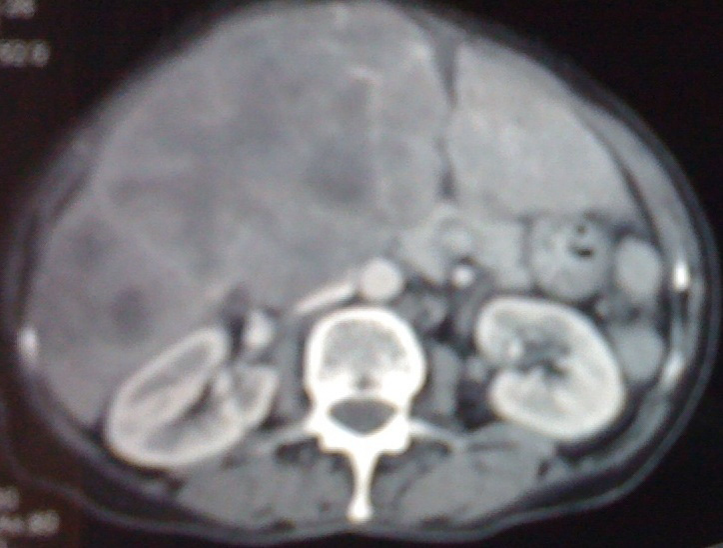
**Liver computed tomography showing multiple diffuse metastatic lesions**.

**Figure 2:**
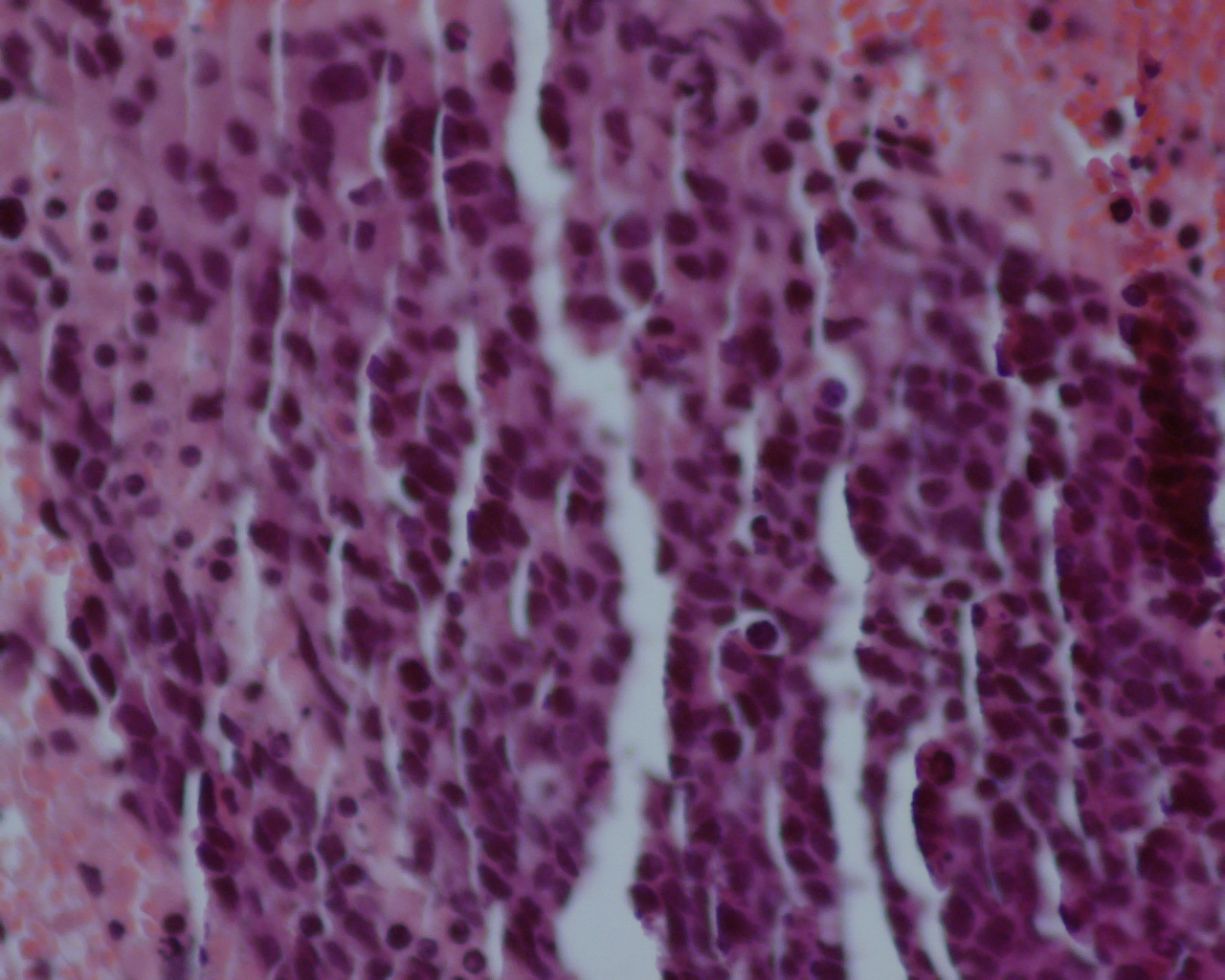
**Photomicrograph of the histopathological analysis of the liver metastases showing the aspect of a neuroendocrine carcinoma**.

**Figure 3:**
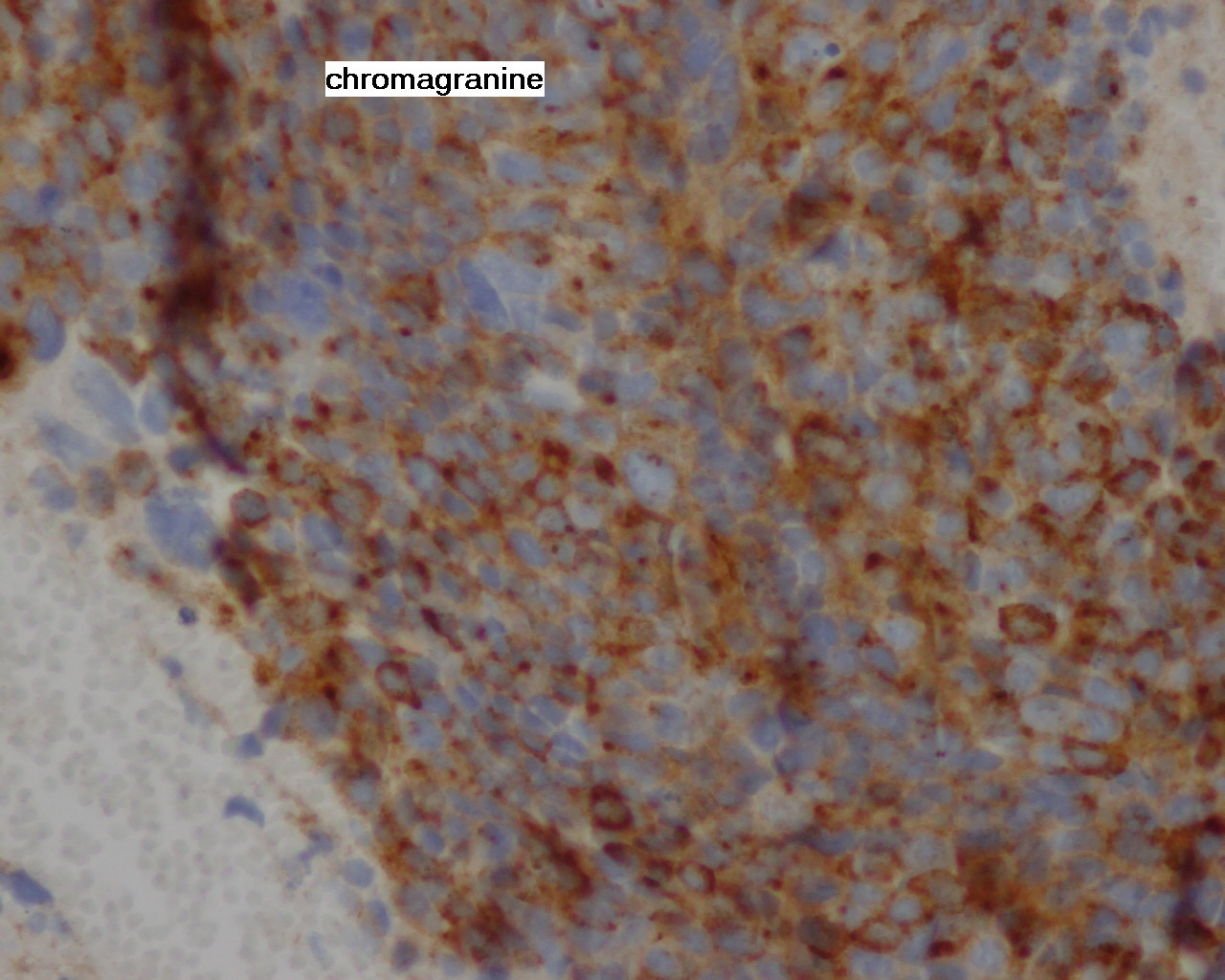
**Chromogranin staining of the liver specimen**.

**Figure 4:**
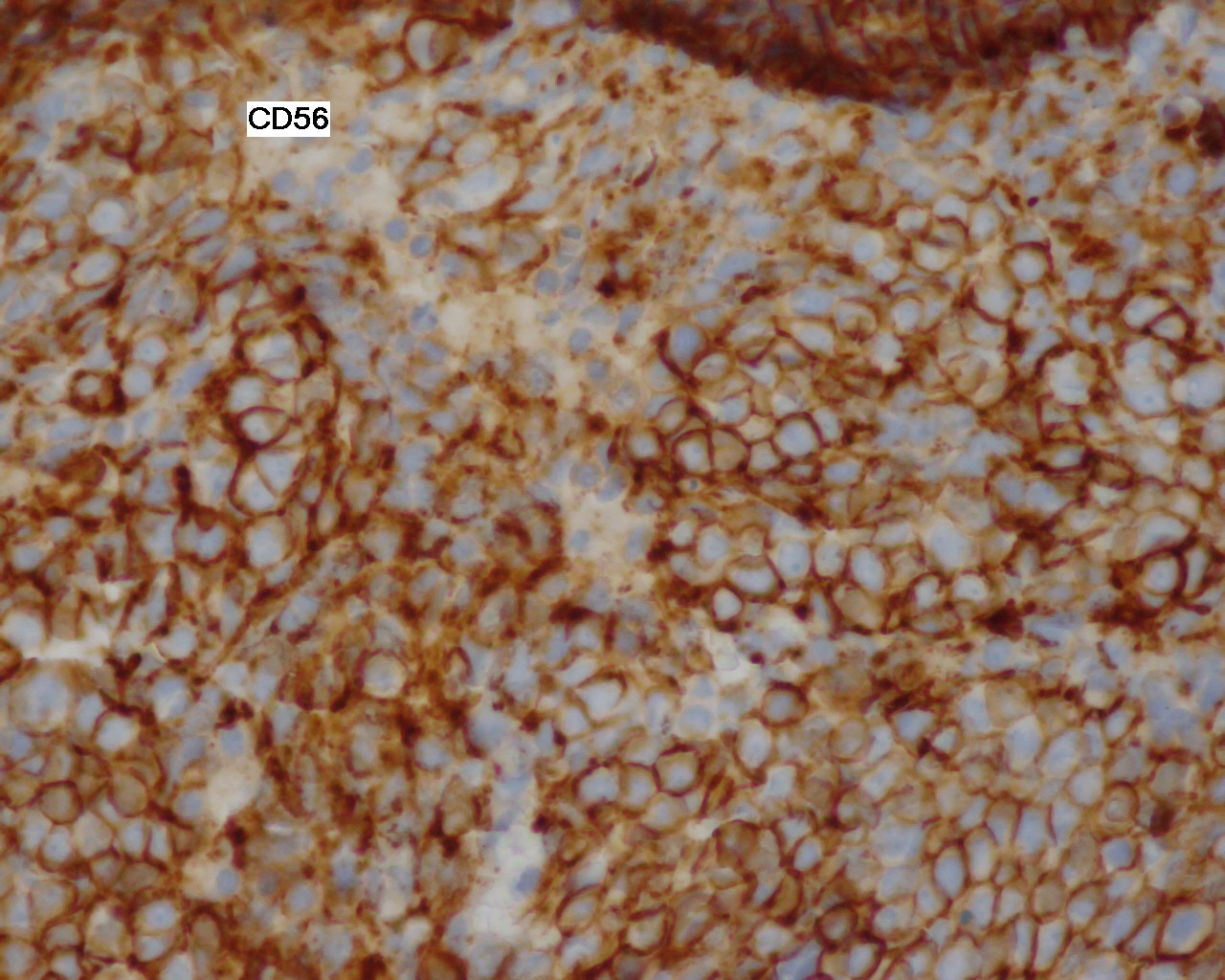
**CD56 staining of the liver specimen**.

Our patient remained in good control and free from relapsed disease over 17 years. At the age of 78 years, she presented with a rapid worsening of performance status. Gynecologic examination didn't reveal any recurrent disease or recurrent skin lesion. However, systematic liver ultrasounds showed multiple diffuse nodular lesions. We performed a computed tomography of her thorax, abdomen and pelvis. Imaging showed diffuse metastatic nodules of her lungs and liver; there was no evidence of relapsing pelvic tumor (Figure 1). We performed a liver biopsy which revealed a histological diagnosis of liver metastases from a neuroendocrine carcinoma, confirmed by immunohistochemistry study with chromogranin and CD56 positive staining (Figure 2, Figure 3 and Figure 4). There was no clinical evidence of the primary site.

Our patient is undergoing a palliative chemotherapy based on a combination of cisplatin and etoposide.

## Discussion

An association of multiple cancers in a single patient suggests common etiological factors, and our case reveals two principal points of discussion; the first one concerns the relationship between the skin and cervical tumors, and the second one concerns the origin of the neuroendocrine metastatic disease.

Skin metastases from cervical carcinoma count for less than 2% [4]; they are correlated with an advanced disease, multiple metastases dispread and poor prognosis. In the literature there are four reported cases of cervical cancer with metastases to the nose dorsum [5]. In the case that we present, the histological type of the skin lesion as a basal cell carcinoma and the long survival after the local control of both tumors might exclude the hypothesis of skin metastases from the cervical carcinoma. Furthermore, both tumors were diagnosed at the same time suggesting a synchronous association of two primary sites. Baykal *et al*. have already reported a case of four malignancies in the same patient including a cervical carcinoma and a basal cell carcinoma but in a metachronous setting [6].

Human papilloma virus (HPV) infection has a well-established role in the carcinogenesis of cervical squamous cell carcinomas, and many studies have speculated that HPV infection could be involved in the pathogenesis of the nonmelanoma skin cancers (NMSC). R Corbalán-Vélez *et al*. published a review of different studies evoking the involvement of *Epidermodysplasia verruciformis*- associated HPV in the carcinogenesis of skin squamous cancers [7]. Levi *et al*. in the Vaud cancer registry have reported an elevated risk of developing second NMSC in women with cervical, vulvar and vaginal carcinomas. This finding was interpreted to demonstrate the role of HPV in the etiology of NMSC [8]. Hennig *et al*., in a study involving women with HPV16 positive high grade cervical intraepithelial dysplasia (CINIII), found one case of a second basal cell carcinoma of the skin with detected HPV16 in the tumor [9].

Patients with two synchronous or metachronous tumors have a higher risk of developing further malignancies [3]. In a review covering 20 years at the Ellis Fischel State Cancer Hospital, Spratt *et al*. suggested that, on the basis of the observed age-specific incidence cancers, persons living to extreme age can expect to have multiple cancers with great frequency [1]. Our patient developed neuroendocrine carcinomas of unknown primary site 17 years after the initial diagnosis of the synchronous described malignancies.

Neuroendocrine tumors from an unknown primary site are uncommon; they arise from an occult or clinically undetectable primary site in one of several locations (bronchus, pancreas, stomach, colon, rectum and several other sites) [10]. Immunohistochemical studies are useful for the identification of a neoplasm showing neuroendocrine differentiation. The standard panel is synaptophysin, chromogranin and neural cell adhesion molecules (CD56) [11].

The association of basal cell carcinoma and neuroendocrine carcinoma of the skin, also called Merkel cell carcinoma (MCC), is a rare occurrence; however, it is documented [12]. Koljonen *et al*. reported in a recent study that among 172 patients diagnosed with MCC, a total of 11 cases of basal cell carcinoma were detected (standardized incidence ratio, 3.48; 95% CI [1.74-6.22]) [13]. Recently, a new human polyoma virus has been identified in MCC. The new entity was called Merkel cell polyomavirus (MCV); Feng *et al*. reported that MCV sequences were detected in 80% of MCC tumors [14]. In another recent study concerning the detection of MCV sequences in NMSC from immunosuppressed and immunocompetent patients, Kassem *et al*. found that 37.5% of sporadic basal cell carcinoma in immunocompetent patients were MCV positive [15].

Metastases could be from a MCC component undetected initially on the skin lesion. However, MCC is an aggressive skin cancer and the long survival of our patient and the absence of recurrence within several years may eliminate this hypothesis. Otherwise, occult neuroendocrine component of a cervical tumor diagnosed initially as squamous carcinoma, and responsible for ulterior metastasis, has been already reported in the literature [16]; this might be an explanation for the evolution of the disease in our patient.

Other interpretations of this association of multiple cancers that we report could be hereditary factors and genetic predisposition, but we do not have information about the familial history of our patient. Otherwise this unusual association could be due to a chance phenomenon.

## Conclusions

MPCs occur rarely. The etiology remains controversial and a large number of cancer patients have to be followed for long periods to obtain adequate data about the development of subsequent additional malignancies.

## Consent

Written informed consent was obtained from our patient for publication of this case report and any accompanying images. A copy of the written consent is available for review by the Editor-in-Chief of this journal.

## Competing interests

The authors declare that they have no competing interests.

## Authors' contributions

MM was involved in the analysis of the data and the literature research, and he also wrote the manuscript. SB helped with the patient management and revision of the manuscript. TM helped with the literature research. RA helped with the literature research. NI helped with modifications and revision of the manuscript. MG helped with the analysis of the data. HE approved the treatment and analyzed the literature data. All authors read and approved the final manuscript.
